# miR-23b-3p Ameliorates LPS-Induced Pulmonary Fibrosis by Inhibiting EndMT via DPP4 Inhibition

**DOI:** 10.1007/s12033-023-00992-9

**Published:** 2023-12-27

**Authors:** Linlin Yue, Feng Chen, Xin Liu, Chaoyu Wu, Jie Wang, Jiying Lai, Hongquan Zhu

**Affiliations:** 1 Department of Intensive Care Unit, The First Affiliated Hospital of Gannan Medical University, Ganzhou, 341000 Jiangxi People’s Republic of China; 2 Department of Pediatric Surgery, The First Affiliated Hospital of Gannan Medical University, Ganzhou, 341000 Jiangxi People’s Republic of China; 3 The First Affiliated Hospital of Gannan Medical University, 128 Jinling Avenue, Zhanggong District, Ganzhou, 341000 Jiangxi People’s Republic of China

**Keywords:** miR-23b-3p, DPP4, Pulmonary fibrosis, EndMT, LPS

## Abstract

Acute respiratory distress syndrome is a disease triggered by severe pulmonary and systemic inflammation that may lead to fibrosis and the decline of lung function. Lung capillary endothelial-to-mesenchymal transition (EndMT) is one of the primary sources of fibroblasts in pulmonary fibrosis. The role of miRNAs as molecular markers of pulmonary fibrosis, and miRNAs as nucleic acid drugs has attracted increasing attention. To mock EndMT process, Human pulmonary microvascular endothelial cells (HPMECs) were induced with lipopolysaccharide (LPS). Similarly, LPS treatment was used to generate a mouse model of LPS-induced EndMT and pulmonary fibrosis. LPS-induced EndMT in HPMECs resulted in a significant reduction of miR-23b-3p. miR-23b-3p inhibited the interstitial transition of HPMECs, and miR-23b-3p could mediate this process via inhibiting dipeptidyl peptidase-4 (DPP4). Dual-luciferase assays confirmed the regulatory mechanism of miR-23b-3p. In our mouse model of LPS-induced pulmonary fibrosis, miR-23b-3p and a DPP4 inhibitor (sitagliptin) individually alleviated LPS-induced EndMT progression and pulmonary fibrosis, and their combined use achieved the strongest remission effect. To sum up, miR-23b-3p alleviates EndMT in pulmonary fibrosis by inhibiting the expression of DPP4.

## Introduction

Acute lung injury [1] and acute respiratory distress syndrome (ARDS) are diseases with high morbidity and mortality rates worldwide. These diseases often develop into pulmonary fibrosis in the late stages, manifesting as severe hypoxemia and accelerating patient death [2]. No effective drugs was curative for ARDS and pulmonary fibrosis up till the present moment.

Pulmonary fibrosis cells are mainly derived from stromal fibroblasts, blood-derived fibroblasts, and fibroblasts from the epithelial-to-mesenchymal transition (EMT) and endothelial-to-mesenchymal transition (EndMT) [3, 4]. EndMT promotes pulmonary fibrosis mainly by transforming pulmonary microvascular endothelial cells into mesenchymal cells, which further differentiate into fibroblasts or myofibroblasts. The loss of endothelial cell markers, such as E-cad and PECAM-1/CD31 and the acquisition of the mesenchymal cell markers, such as α-SMA and FSP1/S100A4 occurred in EndMT [5].

Micro RNAs (miRNAs) are small fragments of RNA, usually 20–25 nucleotides long, that serve to degrade their target mRNAs by binding to complementary sequences within their targets [6]. miRNAs are involved in EMT and EndMT. For example, overexpression of miR-449a was found to inhibit E-cadherin expression while upregulating α-SMA expression [7]. miR-374b induces EndMT by regulating the MAPK signaling pathway [8]. miR-15a, miR-23b, and miR-199a act as inhibitors of EndMT progression in atrioventricular canal (AVC) development [9]; however, miR-199a promotes EndMT in umbilical vein endothelial cells (HUVECs) [10]. Thus, the same miRNA may play different roles in EndMT in different cell types. This highlights the need for further investigation of miRNA function in pulmonary fibrosis.

Dipeptidyl peptidase-4 (DPP4) is a serine protease expressed in injured skin, the central nervous system, placenta, fat, muscle, and lung fibroblasts [1, 11]. DPP4 inhibitors can promote insulin release from islet β-cells and are common drugs for the treatment of type 2 diabetes [12]. Recently, several studies have exhibited that DPP4 expression is elevated in fibroblasts in skin lesions, and DPP4 inhibitors can prevent scarring [13]. Soare et al. found that DPP4 acts as a marker of fibroblast activation in systemic sclerosis [14]; Nonetheless, the role of DPP4 in pulmonary fibrosis and the potential therapeutic effects of DPP4 inhibitors in this context have not been studied.

We identified a miRNA, miR-23b-3p, that can alleviate pulmonary fibrosis in an LPS-induced pulmonary fibrosis model. We ascertained that miR-23b-3p can inhibit EndMT in pulmonary fibrosis by targeting DPP4. Simultaneously, a mouse model of LPS-induced pulmonary fibrosis was used to demonstrate in vivo that combined treatment with miR-23b-3p and DPP4 inhibitors can greatly alleviate pulmonary fibrosis in mice.

## Materials and Methods

### Cell Culture and Cell Transfection

The HPMEC line was purchased from PromoCell and cultured in PromoCell Cell Growth Medium at 37 °C. The DPP4 inhibitor (sitagliptin) was purchased from Selleck Chemicals. When the cells reached 70% confluence, RNA was transfected into the HPMECs and 293T cells using Lipofectamine®3000 (Invitrogen). Overexpression and inhibition of miR-23b-3p were achieved by transfection with 20 nM of miR-23b-3p mimic (shbio, China) or miR-23b-3p inhibitor (shbio) into HPMECs. Corresponding scrambled oligonucleotide sequences (mimic-NC or inhibitor-NC, shbio) were used as negative controls.

### RT-qPCR

cDNA from total RNA of HPMECs as a template, GoTaq® qPCR Master Mix (Promega, USA) was used for RT-qPCR analysis using the Applied Biosystems®7500 Real-Time PCR system (California, USA), according to the manufacturer’s instructions. The primers are presented in Table 1.

**Table 1:** RT-qPCR primer sequences

	5′–3′ forward primer sequence	5′–3′ reverse primer sequence
hsa-miR-23b-3p	ACACTCCAGCTGGGATCACATTGCCAGGGATTACCAC	CTCAACTGGTGTCGTGGA
mmu-miR-23b-3p	ACACTCCAGCTGGGATCACATTGCCAGGGATTACC	CTCAACTGGTGTCGTGGA
hsa-miR-15a-5p	ACACTCCAGCTGGGTAGCAGCACATAATGGTTTGTG	CTCAACTGGTGTCGTGGA
hsa-miR-199a-5p	ACACTCCAGCTGGGCCCAGTGTTCAGACTACCTGTTC	CTCAACTGGTGTCGTGGA
hsa-miR-218-5p	ACACTCCAGCTGGGTTGTGCTTGATCTAACCATGT	CTCAACTGGTGTCGTGGA
hsa-miR-302c-3p	ACACTCCAGCTGGGTAAGTGCTTCCATGTTTCAGTGG	CTCAACTGGTGTCGTGGA
hsa-miR-192-5p	ACACTCCAGCTGGGCTGACCTATGAATTGACAGCC	CTCAACTGGTGTCGTGGA
hsa-miR-194-5p	ACACTCCAGCTGGGTGTAACAGCAACTCCATGTGGA	CTCAACTGGTGTCGTGGA
Universal stem-loop reverse transcription	CTCAACTGGTGTCGTGGAGTCGGCAATTCAGTTGAGGAAAAACGC	
U6	CTCGCTTCGGCAGCACAT	TTTGCGTGTCATCCTTGCG
GAPDH	GTCTCCTCTGACTTCAACAGCG	ACCACCCTGTTGCTGTAGCCAA
DPP4 (human)	AAAGGCACCTGGGAAGTCATCG	CAGCTCACAACTGAGGCATGTC
DPP4 (mouse)	CACCTCTGATGGAAGCAGCTTC	CGAAGCTCTGACCAGCGATTATC
CD31 (human)	GCATATCCAAGGTCAGCAGC	ATGGAGCAGGACAGGTTCAGTC
CD31 (mouse)	CCAAAGCCAGTAGCATCATGGTC	GATGGTGAAGTTGGCTACAGG
VE-cad (human)	GAAGCCTCTGATTGGCACAGTG	TTTTGTGACTCGGAAGAACTGGC
VE-cad (mouse)	GAACGAGGACAGCAACTTCACC	GTTAGCGTGCTGGTTCCAGTCA
α-SMA (human)	CTATGCCTCTGGACGCACAAC	CAGATCCAGACGCATGATGGCA
α-SMA (mouse)	TGCTGACAGAGGCACCACTGAA	CAGTTGTACGTCCAGAGGCATAG
S100A4 (human)	CAGAACTAAAGGAGCTGCTGACC	CTTGGAAGTCCACCTCGTTGTC
S100A4 (mouse)	AGCTCAAGGAGCTACTGACCAG	GCTGTCCAAGTTGCTCATCACC

### Western Blotting (WB)

30 µg of denatured proteins from HPMECs and 293T cells were subjected to 10% SDS-PAGE, incubated overnight with primary antibodies at 4 °C. The primary antibodies used were CD31 (1:1000, ab9498, abcam), VE-cad (1:1 000, ab205336, abcam), α-SMA (1:1 000, ab5694, abcam), S100A4 (2 µg/mL, ab218511, abcam), and DPP-4 (1:1000, 67138, CST) incubated with goat anti-rabbit (1:10,000, SA00001-2, Proteintech) or goat anti-mouse (1:10,000, SA00001-1, Proteintech) secondary antibodies. GAPDH was used as the loading control (1:10 000,10494-1-AP).

### Immunofluorescence Staining

HPMECs were grown on coverslips to 40–50% confluency, fixed in 4% formaldehyde for 20–30 min and permeabilized with 0.1% Triton X-100 for 2–5 min, incubated with primary antibodies (diluted with 1% BSA) in a humidified chamber at 4 °C overnight. The primary antibodies used were CD31 (1 µg/mL, ab9498, abcam), VE-cad (1:5 00, ab205336, abcam), α-SMA (1:200, ab5694, abcam), S100A4 (2 µg/mL, ab218511, abcam), and DPP4 (1:200, 67138, CST). Cells were then incubated with either 488-conjugate (1:1000, SA00013-2, proteintech) or 594-conjugated secondary antibody (1:1000, SA00013-4, proteintech) for 30 min. Nuclei were stained with DAPI.

#### Dual-Luciferase Assay

DPP4 3′UTR containing the wild-type or mutant binding site of hsa-miR-23b-3p were subcloned into the psiCHECK2 vector (C8021, Promega). psiCHECK2 plasmid containing the insertion sequence and hsa-miR-23b-3p mimic/mimic-negative control (NC) or miR-23b-3p inhibitor/inhibitor-NC were co-transfected into 293T cells by Lipofectamine 2000 (Invitrogen). The activities of *Firefly* and *Renilla* luciferase were measured by the Dual-Luciferase Reporter Assay Kit (E1910, Promega). The sequences used were as follows:

miR-23b-3p mimic: 5′-UCACAUUGCCAGGGAUUACCAC-3′

mimic-NC: 5′-AAUCUAGGCGCCUUAACAGCCUA-3′

miR-23b-3p inhibitor: 5′-GUGGUAAUCCCUGGCAAUGUGAU-3′

inhibitor-NC: 5′-UAAGUCUCUAUGAGAUCGGGCGU-3′

### Animals

A total of 25 8-week-old C57bl/6 specific pathogen-free mice obtained from the Guangdong Medical Laboratory Animal Center were used in this study. Mice were anesthetized by intraperitoneal anesthesia and LPS (6 mg/kg) was instilled into the lungs through the trachea by exposure tracheotomy. In this study, a mouse model of endotoxin-induced acute lung injury was established. 48 h after airway instillation of LPS, the mice were sacrificed and lung tissue was collected. Three independent and blinded histopathologists evaluated the tissue sections under light and fluorescence microscopy. The Animal Care and Use Committee of Gannan Medical University approved the animal experiments used in this study.

### Hematoxylin and Eosin (H&E) Staining

4 μm Paraffin sections were dewaxed once in xylene, graded ethanol and stained with H&E dye (C0105S, beyotime) at room temperature for 3–5 min. Sections were then rinsed with running water, dehydrated, air-dried, and observed under a microscope.

### Masson’s Trichrome Staining

4 μm paraffin sections were dewaxed once in xylene, graded ethanol and stained with hematoxylin dye for 5–10 min in graded alcohol, then stained with Masson’s trichome (HT15, sigma) stain for 5–10 min, then soaked for 30 s, differentiated for 3–5 min, stained with aniline blue for 5 min, soaked for 30 s, dehydrated.

### Immunohistochemistry

4 μm paraffin sections were dewaxed once in xylene, gradient ethanol and antigen retrieval for 20 min in 0.01 M sodium citrate. Sections were then incubated with 3% H_2_O_2_ solution for 10 min, blocked and incubated overnight at 4 °C with primary antibody [CD31 (0.5 µg/mL, ab9498, abcam), α-SMA (1:100, ab5694, abcam), DPP-4 (1:100, 67138, CST)], incubated with secondary antibody (1:2000, SA00001-2, Proteintech) at 37 °C for 30 min, developed in 3,3*N*-Diaminobenzidine Tertrahydrochloride (DAB) solution (P0203, beyotime) for 3 min, stained, differentiated, dehydrated.

### Statistical Analyses

Data are expressed as the mean ± standard deviation. One-way analysis of variance (ANOVA) and Dunnett’s post hoc test were performed for comparisons between more than two groups of data, and the Student’s *t*-test was used to analyze the means of two groups. The threshold for statistical significance was set at *P* < 0.05. All experiments were repeated three times.

## Results

### miR-23b-3p is Lowly Expressed During EndMT

**Fig. 1:**
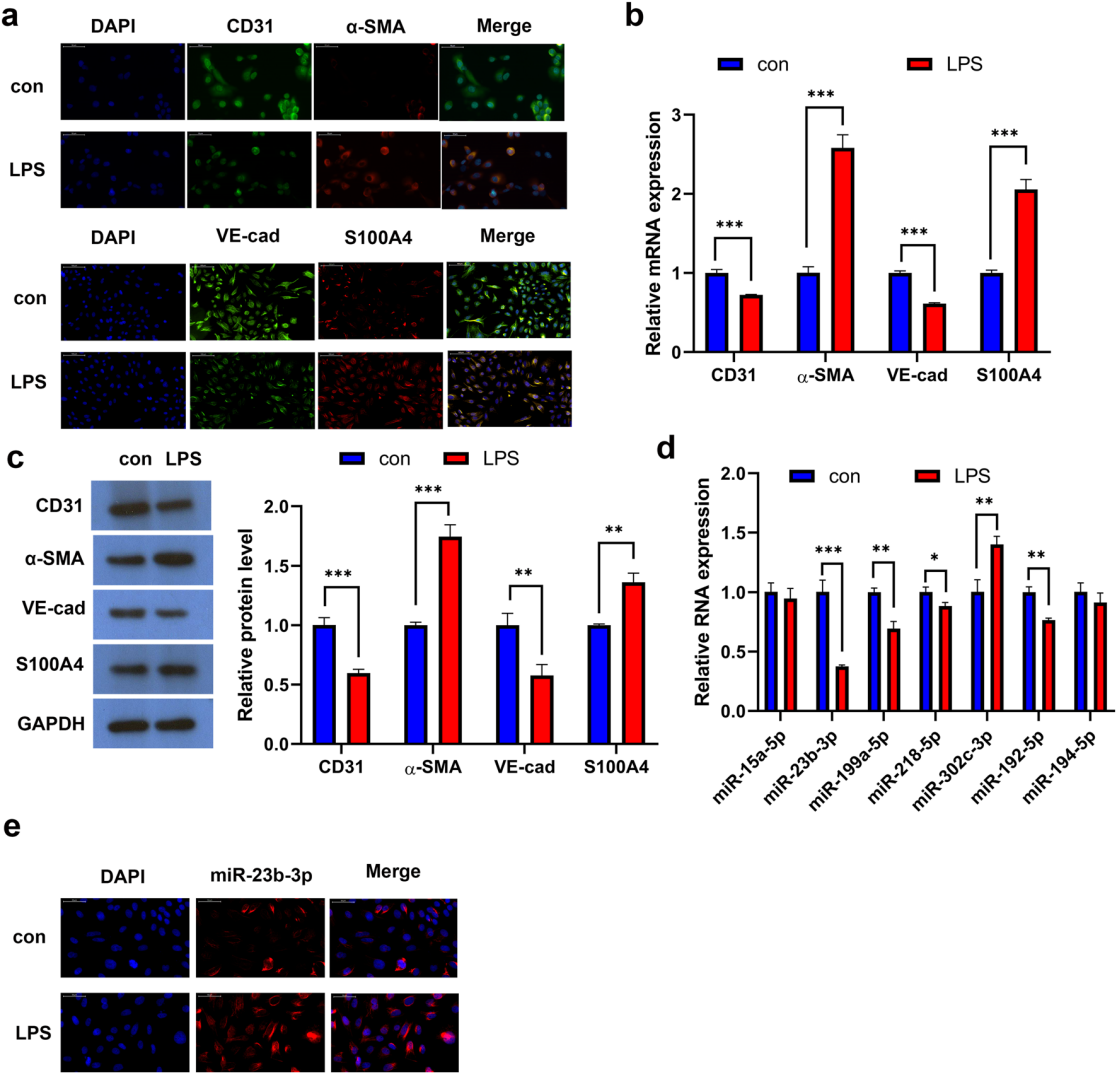
Low expression of miR-23b-3p in HPMECs during EndMT **a** Immunofluorescence of CD31, VE-cad, α-SMA, and S100A4 in LPS-treated and untreated HPMECs. qPCR (**b**) and WB (**c**) detection of CD31, VE-cad, α-SMA, and S100A4 expression in LPS-treated and untreated HPMEC cells. **d** RT-qPCR detection of EMT/EndMT process-related microRNAs. **e** Immunofluorescence of miR-23b-3p in LPS-treated and untreated HPMEC cells

Echeverria et al. previously reported that treatment with 20 μg/mL LPS induces fibrosis in endothelial cells [15]. We also found that the endothelial markers CD31 and VE-cad were downregulated, while the expression of the mesenchymal markers α-SMA and S100A4 were increased in HPMECs with 20 μg/mL LPS (Fig. 1a–c). Therefore, LPS promoted the mesenchymal transition of HPMECs.

It is universally acknowledged that miRNAs play important roles in EMT and EndMT. In particular, microRNAs miR-15a-5p, miR-23b-3p, miR-199a-5p, miR-218-5p, miR-302c-3p, miR-192-5p, and miR-194-5p have been reported to inhibit EndMT in LPS-induced EndMT models [9, 16–18]. Thus, we investigate the expression of these miRNAs in LPS-treated HPMECs. Our qPCR results demonstrated that miR-23b-3p was immensely down-regulated in LPS-treated HPMECs (Fig. 1d). Furthermore, immunofluorescence was able notice low miR-23b-3p expression in LPS-treated HPMECs (Fig. 1e).

### miR-23b-3p Reverses LPS-Induced EndMT Progression in HPMECs

**Fig. 2:**
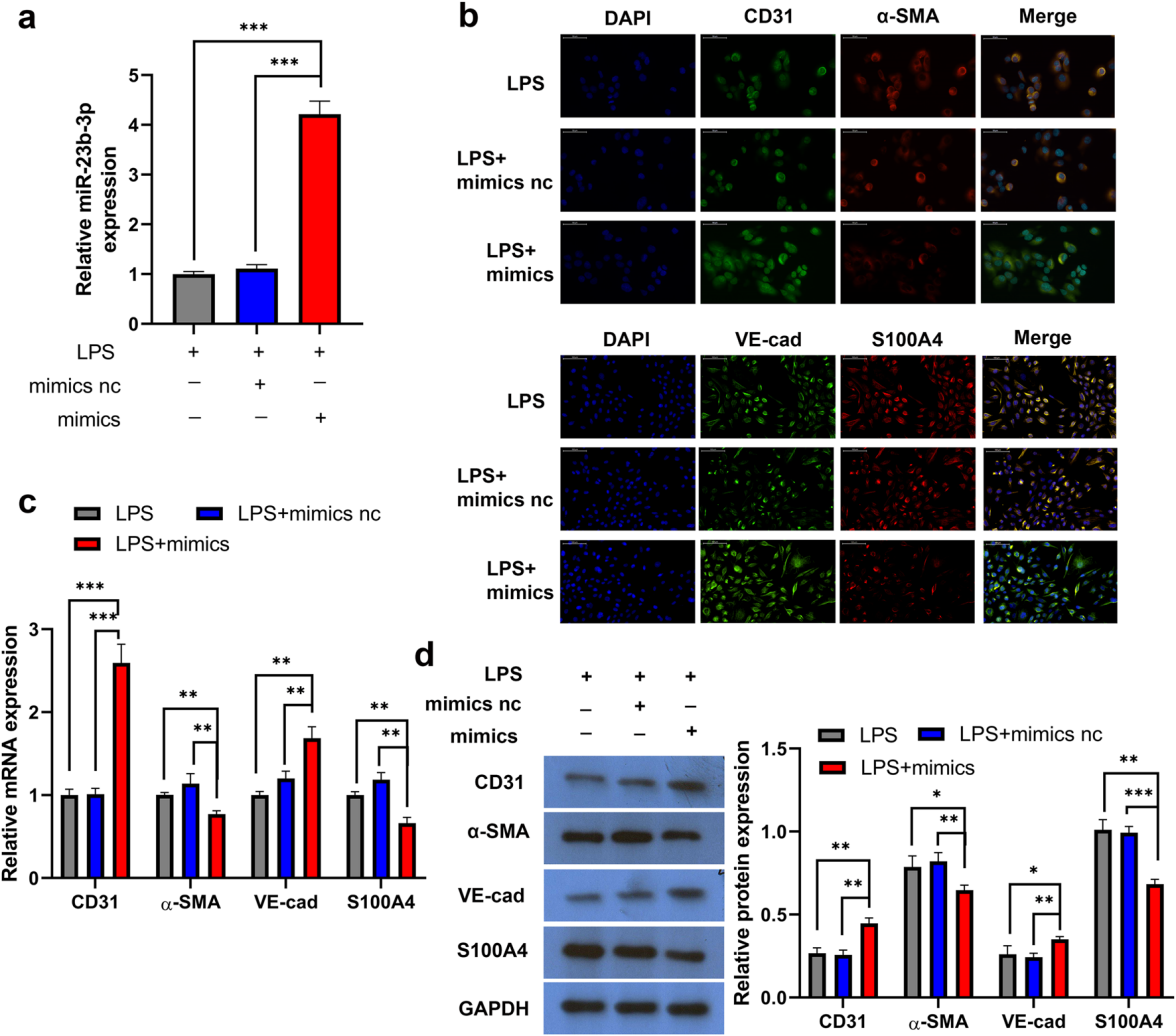
miR-23b-3p reverses LPS-induced EndMT in HPMECs Immunofluorescence (**a**), qPCR (**b**) and WB (**c**) detection of CD31, VE-cad, α-SMA, and S100A4 in LPS-treated HPMECs with miR-23b-3p mimic or mimic-NC

LPS-induced HPMECs were transfected with the miR-23b-3p mimic or scrambled oligonucleotide negative control sequences (mimic-NC), miR-23b-3p was successfully vastly expressed in LPS-induced HPMECs, and mimics-NC had no significant effect on the expression of miR-23b-3p (Fig. 2a). The results of immunofluorescence, RT-qPCR, and WB all demonstrated that the miR-23b-3p mimic rised the expression of CD31 and VE-cad (endothelial cell markers) in LPS-induced HPMECs, and lessend LPS-induced expression of α-SMA and S100A4 (the mesenchymal markers) in HPMECs (Fig. 2b–d). In sum, the above results prescribed that miR-23b-3p reversed LPS-induced EndMT progression in HPMECs.

### miR-23b-3p can Alleviate LPS-Induced Pulmonary Fibrosis by Inhibiting EndMT In Vivo

**Fig. 3:**
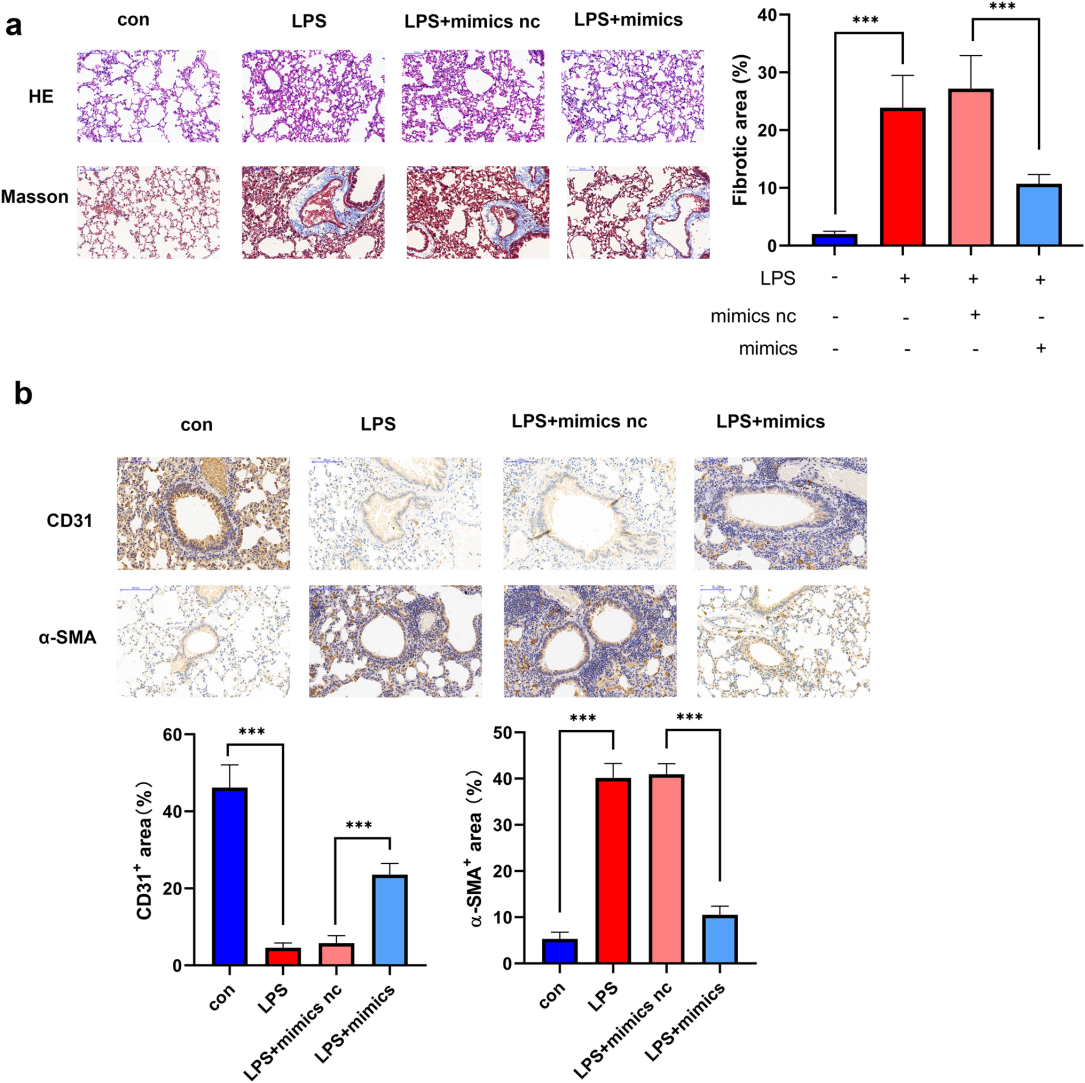
miR-23b-3p can alleviate LPS-induced pulmonary fibrosis by inhibiting EndMT in vivo **a** H&E and Masson’s trichrome staining of mouse lung tissue following tail vein injection of miR-23b-3p agomir in LPS-induced pulmonary fibrosis model in mice. **b** Immunohistochemistry of CD31 and α-SMA in fibrotic mouse lung tissue following tail vein injection of miR-23b-3p agomir in LPS-induced pulmonary fibrosis model in mice, positive area% = positive area/total area

LPS-induced C57 mice were used to simulate an ARDS lung injury model, and miR-23b-3p agomir was injected by the tail vein in LPS-induced mice to study the effect of miR-23b-3p on ARDS-induced pulmonary fibrosis. H&E staining showed LPS-induced alveolar size heterogeneity as well as significant immune cell infiltration, which was significantly alleviated by miR-23b-3p agomir. Masson staining showed that interstitial and small peribronchiolar tissue fibrosis was evident in LPS-induced ARDS mice, comparing with the untreated group, and miR-23b-3p significantly inhibited fibrosis around lung bronchioles (Fig. 3a). Subsequently, the expression of endothelial marker CD31 and mesenchymal marker α-SMA was detected in LPS-induced ARDS mice to investigate the effect of miR-23b-3p on EndMT processes in lung tissue during ARDS-induced pulmonary fibrosis. Immunohistochemical staining revealed that miR-23b-3p boosted the expression of CD31 and diminished the expression of α-SMA in LPS-induced pulmonary fibrosis tissue (Fig. 3b). Therefore, miR-23b-3p blocked EndMT in LPS-induced pulmonary fibrosis tissue.

### miR-23b-3p Targets DPP4

**Fig. 4:**
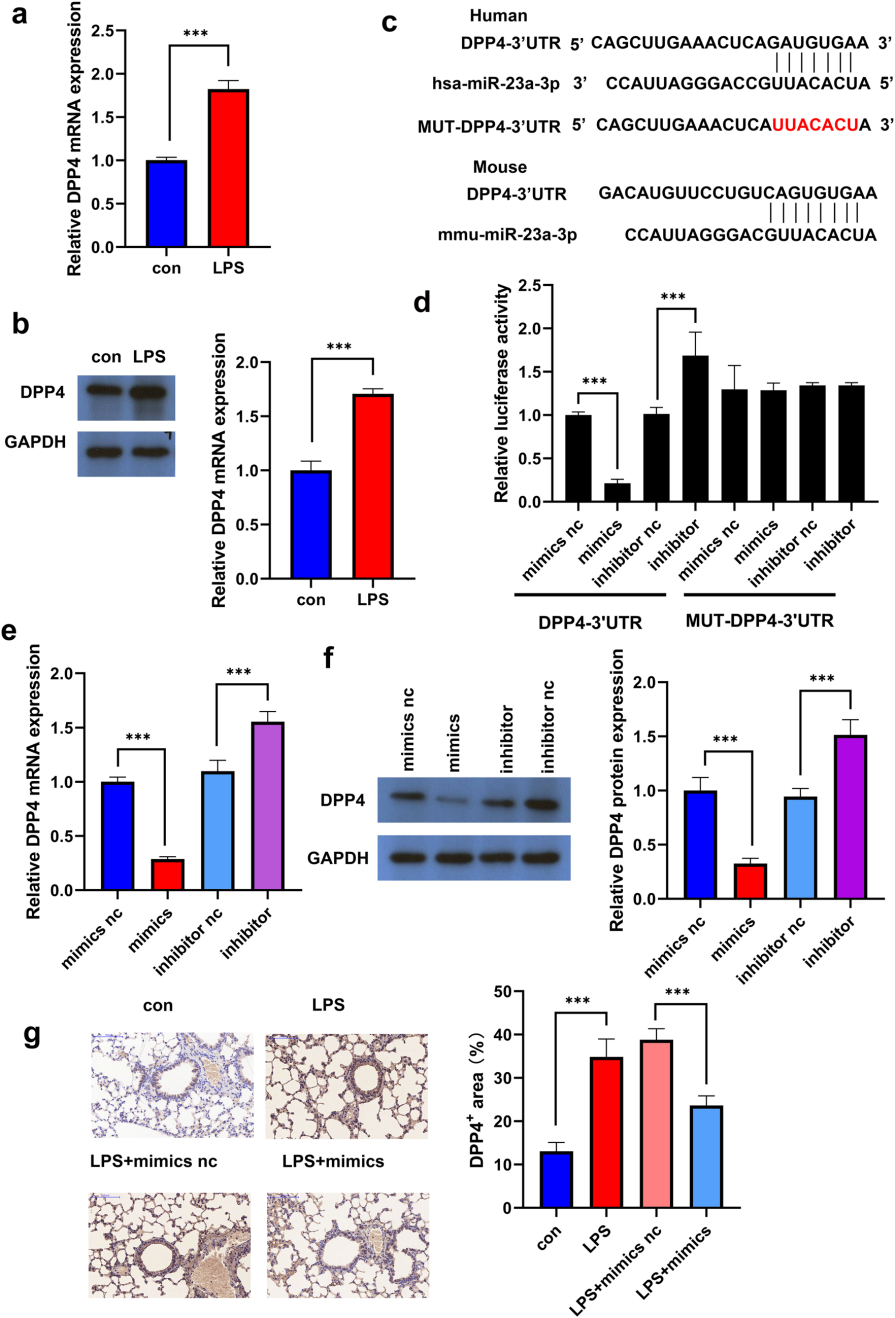
miR-23b-3p targets DPP4 RT-qPCR (**a**) and WB (**b**) of DPP4 expression. **c** Targetscan and BiBiServ software predictions of the binding region of miR-23b-3p to the 3′UTR of DPP4 mRNA and binding region conservation across species. **d** Dual-luciferase assay of the binding of miR-23b-3p to DPP4. RT-qPCR (**e**) and WB (**f**) detection of DPP4 expression following treatment with miR-23b-3p. **g** Immunohistochemical detection of DPP4 expression in fibrotic mouse lung tissue with or without miR-23b-3p agomir treatment in LPS-induced pulmonary fibrosis model in mice, positive area% = positive area/total area

DPP4 is a serine exopeptidase ubiquitously expressed on the surface of cell membranes. Multiple studies have shown that DPP4 inhibitors improve pulmonary fibrosis [19, 20]. In our study, DPP4 was highly expressed in LPS-treated HPMECs at the transcriptional and translational level (Fig. 4a, b). TargetScan and BiBiServ software predicted that miR-23b-3p might target the 3′UTR of DPP4 mRNA, as it’s binding sites were quite conserved in humans and mice (Fig. 4c). Indeed, dual-luciferase experiments confirmed the 3′UTR of DPP4 mRNA targeting by miR-23b-3p in 293 cells (Fig. 4d). miR-23b-3p partially silenced the expression of DPP4, further supporting DPP4 as a target of miR-23b-3p (Fig. 4e, f). Concurrently, the expression of DPP4 was gained in LPS-induced pulmonary fibrosis blood vessels and surrounding tissues in mice, and miR-23b-3p could partially restrained the expression of DPP4 in mouse pulmonary fibrosis blood vessels and surrounding tissues (Fig. 4g).

### DPP4 Inhibition Rescues miR-23b-3p Inhibition and Reverses LPS-Induced EndMT

**Fig. 5:**
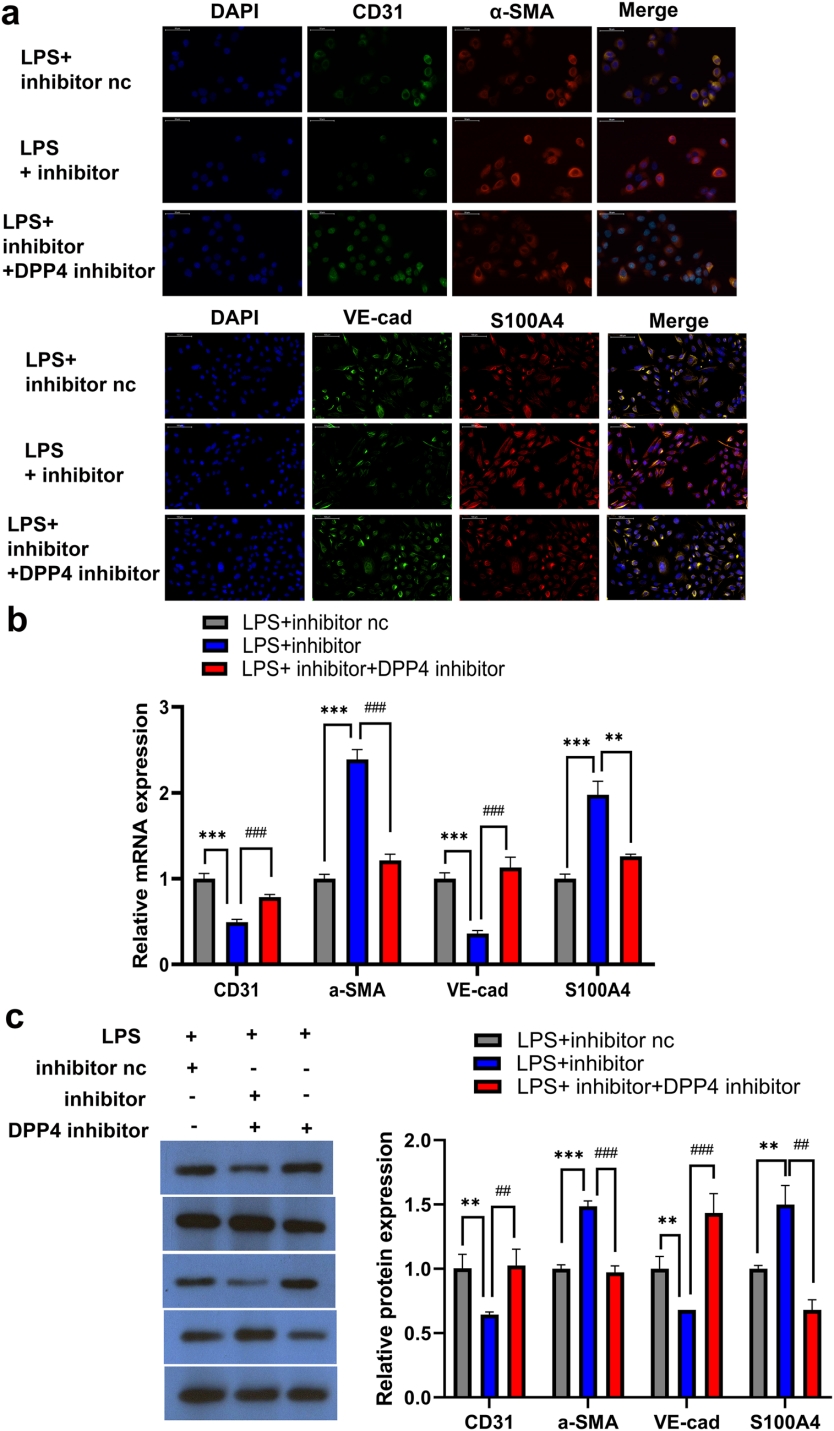
DPP4 inhibition rescues miR-23b-3p inhibition and reverses LPS-induced EndMT Immunofluorescence (**a**), RT-qPCR (**b**) and WB (**c**) of CD31, VE-cad, α-SMA, and S100A4 in LPS-treated HPMECs treated with miR-23-3p inhibitor and the DPP4 inhibitor, sitagliptin

LPS-treated HPMECs with a miR-23b-3p inhibitor promoted changes in gene expression consistent with the progression of EndMT. To jude the impact of DPP4 in EndMT, we treated LPS-induced HPMECs transfected with miR-23b-3p inhibitor with 0.5 μM of the DPP4 inhibitor, sitagliptin. HPMECs with sitagliptin rescued the effect of miR-23b-3p inhibition by raising the expression of CD31 and VE-cad and impairing the expression of α-SMA and S100A4 in LPS-treated HPMECs (Fig. 5a–c). Therefore, DPP4 inhibitors may represent a promising therapeutic direction for treating EndMT-related diseases, including pulmonary fibrosis.

### The Combination of miR-23b-3p and DPP4 Inhibitor is More Beneficial to Alleviate LPS-Induced Pulmonary Fibrosis

**Fig. 6:**
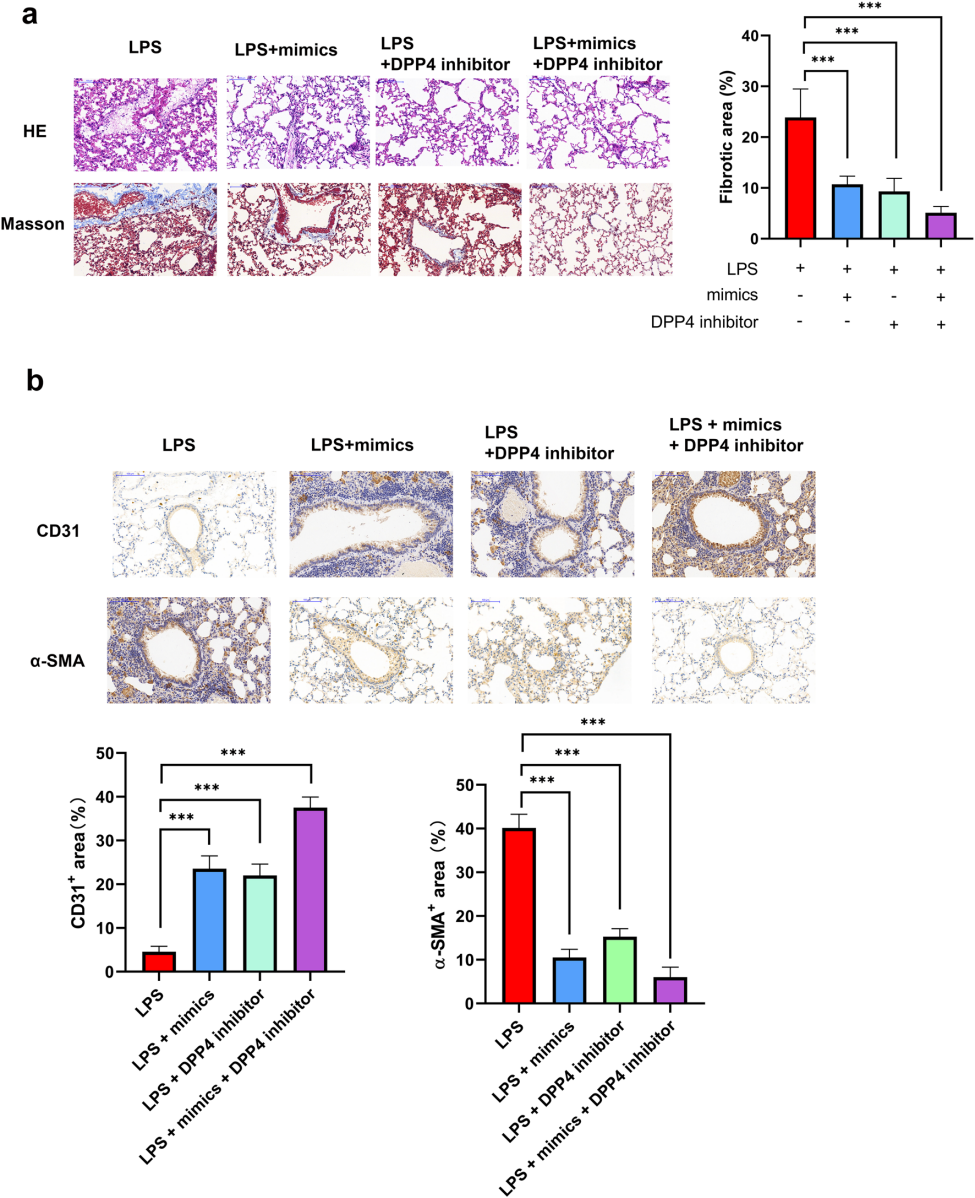
miR-23b-3p can alleviate LPS-induced pulmonary fibrosis by inhibiting EndMT **a** H&E and Masson’s trichrome staining of mouse lung tissue following tail vein injection of sitagliptin alone or in combination with miR-23b-3p agomir in LPS-induced pulmonary fibrosis model in mice. **b** Immunohistochemistry of CD31 and α-SMA in fibrotic mouse lung tissue following tail vein injection of sitagliptin alone or in combination with miR-23b-3p agomir in LPS-induced pulmonary fibrosis model in mice, positive area% = positive area/total area

In order to evaluate whether the combination of DPP4 inhibitor and miR-23b-3p is more conducive to the remission of LPS-induced pulmonary fibrosis in vivo, we injected 100 nM sitagliptin alone or in combination with miR-23b-3p agomir into the tail vein of LPS-induced fibrotic mice. We found that combinatorial treatment with sitagliptin and miR-23b-3p agomir resulted in a stronger inhibitory effect on pulmonary fibrosis than either treatment alone (Fig. 6a). Sitagliptin treatment enhanced the expression of CD31 and lessened the expression of α-SMA in LPS-induced pulmonary fibrosis blood vessels and surrounding tissues in mice, and EndMT was further aggravated by the addition of miR-23b-3p agomir (Fig. 6b). Overall, the combination of miR-23b-3p and DPP4 inhibitor is more beneficial to alleviate LPS-induced pulmonary fibrosis.

## Discussion

Pulmonary fibrosis is a well-recognized sequela of ARDS, and the degree of fibrotic proliferation in ARDS is strongly associated with poor prognosis, increasing patient mortality and ventilator dependence [21]. The mechanisms by which patients with ARDS develop an excessive fibrotic response from initial inflammation to eventual recovery remain unclear [22]. In this study, we used LPS-induced HPMEC cells and LPS-induced mice to mimic the process of inflammation-induced pulmonary fibrosis and to investigate the mechanisms involved.

Our studies revealed the diminution of the endothelial cell markers, CD31 and VE-ca, and enhancement of the mesenchymal markers, α-SMA and S100A4, in LPS-induced HPMECs as they progress through EndMT; this represents the generation of an in vitro model of LPS-induced fibrosis in HPMECs. This is consistent with previous work by Echeverria et al. in which HUVECs were transformed into fibroblasts using LPS [15]. We used our in vitro model to identify miR-23b-3p as having a potentially enormous role in LPS-induced fibrosis, as its expression was significantly inhibited in LPS-induced HPMECs. Consistent with our findings that miR-23b-3p could reverse EndMT in vitro, Yu et al. recently reported that miR-23b-3p inhibits high-glucose-induced renal fibrosis [23]. Thus, miR-23b-3p may be broadly implicated in fibrosis-related diseases.

DPP4 is highly expressed in fibroblasts of patients with systemic sclerosis (SSc), and DPP4 inhibitors alleviated bleomycin-induced pulmonary fibrosis [14]. In our study, the role of miR-23b-3p in reversing EndMT and preventing fibrosis was related to its inhibition of DPP4, as a similar effect was achieved using the DPP4 inhibitor, sitagliptin. This is consistent with previous work by Suzuk et al., who also found that a DPP4 inhibitor, vildagliptin, alleviated LPS-induced pulmonary fibrosis by inhibiting EndMT [20]. Notably, our in vivo experiments using an LPS-induced mouse model of pulmonary fibrosis demonstrated that the combined treatment with miR-23b-3p and sitagliptin enhanced their independent effects to slow down the development of EndMT and pulmonary fibrosis. Thus, we propose that the DPP4 inhibitor, sitagliptin, and miR-23b-3p represent candidate therapeutics that may be used individually or in combination to alleviate pulmonary fibrosis in patients with ARDS.

## Data Availability

The datasets used and analyzed during the current study are available from the corresponding author on reasonable request.

## Abbreviations

LPS: Lipopolysaccharide
EndMT: Endothelial–mesenchymal transition
EMT: Epithelial–mesenchymal transition
DPP4: Dipeptidyl peptidase-4
ARDS: Acute respiratory distress syndrome
PECAM-1/CD31: Platelet endothelial cell adhesion molecule-1
VE-cad: Vascular endothelial-cadherin
α-SMA: Alpha-smooth muscle actin
FSP1/S100A4: Fibroblast-specific protein-1
